# CovFrameNet: An Enhanced Deep Learning Framework for COVID-19 Detection

**DOI:** 10.1109/ACCESS.2021.3083516

**Published:** 2021-05-25

**Authors:** Olaide Nathaniel Oyelade, Absalom El-Shamir Ezugwu, Haruna Chiroma

**Affiliations:** School of Mathematics, Statistics, and Computer ScienceUniversity of KwaZulu-Natal at Pietermaritzburg Pietermaritzburg 3201 South Africa; Department of Computer ScienceFaculty of Physical SciencesAhmadu Bello University 58989 Zaria 810211 Nigeria; Future Technology Research CenterNational Yunlin University of Science and Technology 34883 Douliu 64002 Taiwan

**Keywords:** Image pre-processing, coronavirus, COVID-19, machine learning, deep learning, convolutional neural network, CNN, X-Ray

## Abstract

The novel coronavirus, also known as COVID-19, is a pandemic that has weighed heavily on the socio-economic affairs of the world. Research into the production of relevant vaccines is progressively being advanced with the development of the Pfizer and BioNTech, AstraZeneca, Moderna, Sputnik V, Janssen, Sinopharm, Valneva, Novavax and Sanofi Pasteur vaccines. There is, however, a need for a computational intelligence solution approach to mediate the process of facilitating quick detection of the disease. Different computational intelligence methods, which comprise natural language processing, knowledge engineering, and deep learning, have been proposed in the literature to tackle the spread of coronavirus disease. More so, the application of deep learning models have demonstrated an impressive performance compared to other methods. This paper aims to advance the application of deep learning and image pre-processing techniques to characterise and detect novel coronavirus infection. Furthermore, the study proposes a framework named CovFrameNet., which consist of a pipelined image pre-processing method and a deep learning model for feature extraction, classification, and performance measurement. The novelty of this study lies in the design of a CNN architecture that incorporates an enhanced image pre-processing mechanism. The National Institutes of Health (NIH) Chest X-Ray dataset and COVID-19 Radiography database were used to evaluate and validate the effectiveness of the proposed deep learning model. Results obtained revealed that the proposed model achieved an accuracy of 0.1, recall/precision of 0.85, F-measure of 0.9, and specificity of 1.0. Thus, the study’s outcome showed that a CNN-based method with image pre-processing capability could be adopted for the pre-screening of suspected COVID-19 cases, and the confirmation of RT-PCR-based detected cases of COVID-19.

## I. Introduction

The 2019 novel coronavirus disease presents an important and urgent threat to global health. It has equally exposed the fragility of the most highly placed health institutions and infrastructures across the globe [1], [2]. Since the first recorded case of COVID-19 in early December 2019 in Wuhan, in the Hubei province of the People’s Republic of China, the number of patients confirmed to have contracted the disease has exceeded 35,960,908 in 214 countries, and the number of people infected is probably much higher. Moreover, a record estimate of more than 1,052,310 people have died from the coronavirus COVID-19 outbreak as of October 06, 2020 [58]. The study conducted by Wynants *et al.* [1] and Taiwo and Ezugwu [3] revealed that the implementation of efficient prediction models, which combine several variables or features, can assist medical staff in triaging patients when allocating limited healthcare resources.

Singh *et al.* [4] developed a deep convolution neural network (CNN) that was applied in the automated diagnosis and analysis of COVID-19 in infected patients. Their model involved tuning hyper-parameters of the CNN model with a multi-objective adaptive differential evolution algorithm. The comparative analysis showed that their proposed method outperformed existing machine learning models such as CNN, GA- and PSO-based CNN models, based on the different performance metrics employed to validate the conducted experiment, such as the F-measure and Sensitivity Specificity, and Kappa statistics. A study in [5], [4] developed DenseNet201, a deep transfer machine learning model for the diagnosis and detection of COVID-19 cases from chest (Computed Tomography) CT scans. The DenseNet201 utilized some feature extraction techniques by adopting its own learned weights on the ImageNet dataset along with a convolutional neural structure. The DenseNet201 model achieved a 97% accuracy compared to other models. In the study presented by Barstugan *et al.* [6], a machine learning approach was proposed to detect the COVID-19 on abdominal Computed Tomography (CT) images. The obtained results showed that their model differentiated COVID-19 specific characteristics from other viral pneumonia. In the works of [7] and [8], the authors implemented a deep convolutional neural networks model that was able to detect COVID-19 pneumonia patients using digital chest X-Ray radiographs automatically. The authors in [9] employed a supervised deep learning model to detect and classify COVID-19 infection from CT images while minimizing the requirements for manual labelling of CT images. More so, the model could efficiently distinguish between −ve and +ve cases of COVID-19 by using samples from retrospectively extracted CT images from multi-scanners and multicenters. The experimental results showed that the existing supervised learning model was able to achieve high precision classifications and good qualitative visualization for the lesion detections. For a comprehensive review of existing machine learning models for COVID-19, interested readers are referred to the following references [11], [12], [21].

Although artificial intelligence approaches such as case-based reasoning (CBR) [13], LSTM [61], and sentiment analysis [62] using text-based input have been applied in the detection of the novel coronavirus disease. The CNN model approaches, however, have shown to be more effective and promising. Several studies [14], [15], [16], [17], [18], [19] and [11], [66], [67] on the application of CNN to the task of detecting and classifying COVID-19 have proven that the deep learning model is one of the most popular and effective approaches in the diagnosis of COVID-19 from digitized images. The outstanding performance of CNN is due to its ability to learn features automatically from digital images as has been applied to the diagnoses of COVID-19 based on clinical images, CT scans, and X-Rays of the chest by researchers. Therefore, considering the advantages of the several automated deep learning solutions approaches as mentioned above for curbing the spread of COVID-19 through early detection, classification, isolation and treatment of affected persons, it would be worthwhile to investigate further the possibility of developing better and more efficient variants of deep machine learning techniques. Moreover, we discovered that most studies fell short in hyperparameter selection in the CNN design and other application of image pre-processing techniques, a limitation which this study addresses.

Motivated by the widely reported role of chest X-Rays in enabling the detection of COVID-19 [59], [60], this paper proposes the application of image pre-processing and deep learning techniques to automate the process of extracting important features. The resulting classification or detection from digital images will provide automation of the process of speeding up diagnoses of the SARS-CoV-2 virus and be supportive in overcoming the issue of a shortage of trained physicians in remote communities [20]. The novelty of the new system is based on the multi-layer image processing techniques and stacking of the convolutional-pooling blocks of the CNN architecture, which is capable of obtaining impressive detection accuracy results. In addition, we propose a framework named CovFrameNet, which demonstrates a pipeline of image pre-processing techniques, deep learning model and result verification approach. The proposed model implementation was such that we first applied some selected image pre-processing techniques to reduce the noise on CT and chest X-Rays digital images obtained from the COVID-19 X-Ray dataset. All the datasets used to validate the performance superiority of the new model were taken from the National Institute of Health (NIH) chest X-Ray datasets. Specifically, the technical contributions of this research are summarized as follows:

• Design of a new CNN based deep learning framework consisting of image pre-processing techniques, deep learning model, and result verification mechanism.
• Application of the proposed image pre-processing techniques to the image datasets for further smoothening and denoising.
• Design of an enhanced CNN architecture aimed at detecting COVID-19 cases using chest X-Ray images from COVID-19 chest X-Ray datasets.
• Investigation of the behavior and performance of the proposed CNN architecture using two optimization algorithms, namely Adam and Stochastic gradient descent (SGD).
• Comparative analysis of the enhanced pre-processing based CNN model with existing state-of-the-art results from literature using the following metrics: accuracy, sensitivity, specificity, F1-score, a confusion matrix, and AUC using receiver operating characteristic (ROC).

The rest of the paper is structured as follows: Section II presents an in-depth literature review on COVID-19 related studies. In section III, we detail the proposed deep learning framework for the characterization of coronavirus on chest X-Ray images and datasets. The computational results and different experimentations are reported in section IV. The interpretation of the results obtained is presented in section V, and the limitation of the study is highlighted in section VI. Finally, the concluding remarks and future research direction are given in section VII.

## II. Related Works

This section presents in-depth advances made by researchers in proposing deep learning algorithms in detecting novel COVID-19 based on different approaches. This aims to clearly point out the difference between our proposed approach and the approaches already discussed in the literature. Since the inception of COVID-19, many deep learning algorithms were applied to detect COVID-19; thus, several surveys and experimental studies have been made on the application of computer vision in improving detection of the disease in digital medical images.

For example, [21] conducted an early literature survey on the detection of COVID-19 through machine learning approaches. Different deep learning approaches were discussed in the survey, including CNN variants such as the SqueezNet, mobilenet, Googlenet, VGG, Inception, Xception, Alexnet, Restnet, etc., and challenges were pointed out with suggestions for future works. Similarly, [22] presented a review on the applications of different aspects of artificial intelligence in combatting COVID-19. The artificial intelligent approaches were applied for diagnosing a variety of symptoms and tests, identifying the severity of the COVID-19 patient, image testing and epidemiology. The detection of COVID-19 through CNN based on X-Rays and CT scans were discussed in the paper. Challenges and recommendations for future study were highlighted. Reference [23] discussed an overview of the applications of artificial intelligence in battling the COVID-19 pandemic. Wynants *et al.* [1] presented a critical survey on the diagnosis and prognosis of COVID-19 for early detection of the virus based on different models, including machine learning.

In addition, empirical works on the diagnosis of COVID-19 via medical images, which were not necessarily covered in the reviews discussed in the preceding section based on CNN exist. For example, Alimadadi *et al.* [23] integrated the CNN and LSTM deep learning algorithms to detect COVID-19 through X-Ray images. In the approach, the CNN was applied to extract features while the LSTM performed the task of detecting COVID-19 from the extracted features. Islam *et al.* [24] proposed combining two deep learning algorithms, namely, bidirectional LSTM and CNN, via the transfer learning approach for the detection of COVID-19 through CT scans and X-Ray images. ANN was used in the study for the segmentation of the lung images to get robust features for the diagnosis. The model was found to improve the performance of detecting COVID-19 with an accuracy of 98.70%. However, there is still room for improving the accuracy as it not up to 100%. Aslan [25] proposed multiple CNN and Bayesnet. The study combined multiple pre-trained CNN for the detection of the COVID-19 pandemic. Features from the multiple CNN and correlation-based feature selection were combined. The Bayesnet performed the COVID-19 diagnosis with an accuracy of 97.44%, and it was found that pre-trained CNN outperformed single CNN. Abraham and Nair [26] proposed SqueezeNet for the detection of COVID-19 from CT scan images. The result of the SqueezNet was found to detect the COVID-19 from the CT scan images with an accuracy of 85.03% better than the complex CNN structure.

Polsinelli *et al.* [27] applied CNN for the diagnosis of COVID-19 from X-Ray images. The approach combined learning and a pre-trained CNN encoder for extracting features representation. The proposed approach was found to improve the accuracy of detecting COVID-19 with an accuracy of 95.6%. Shorfuzzaman and Hossain [28] proposed the application of Visual Geometry Group (VGG-16) based fast regions with CNN (R-VGG-16) for the diagnosing of COVID-19 from X-Ray images. The R-VGG-16 was applied to detect COVID-19 from X-Ray images. Results indicated that the R-VGG-16 achieved an accuracy of 97.36% in detecting COVID-19 patients. Shibly *et al.* [20] proposed CNN for the learning of custom filters in a single convolutional layer to identify particular pneumonia. The approach visualized the region of X-Raysalient with a significant effect on the CNN output. The experiment showed that the CNN detected the COVID-19 from X-Rays with an accuracy of 99.80%.

Karthik *et al.* [29] proposed a variant of CNN called ResNet to diagnose the novel COVID-19 virus from CT scans. The RestNet was applied to detect COVID-19 from CT scan images, and it was found to detect COVID-19 with an accuracy of 95.09%. Raajan *et al.* [30] applied grey wolf optimization algorithm to optimize the hyperparameters of CNN architecture to detect COVID-19 patients. In the study, the hyperparameters of the CNN architecture were optimized through grey wolf optimization algorithm to obtain the CNN model used for the detection of COVID-19. The optimized CNN achieved an accuracy of 97.78% in diagnosing COVID-19 from X-Ray images. Goel *et al.* [31] experimented with 9 variants of CNN Inception ResNet V2, ResNeXt-50, Se-ResNeXt-50 AlexNet, DenseNet121, Inception V4, GoogleNet, ResNet-50 and Se-ResNet-50 for the diagnosing of COVID-19 from X-Rays. Results indicated that Se-ResNeXt-50 had the best accuracy of 99.32% compared to the other CNN variants. Hira *et al.* [32] adopted a transfer learning hybrid of 3D and 2D CNN to detect COVID-19 from X-Ray images. The model combined pre-trained VGG16, shallow CNN and depth wise separable convolution layer and spatial pyramid pooling module. The proposed approach was applied to detect COVID-19 from X-Ray images and achieved an accuracy of 96.91%. Similarly, Bayoudh *et al.* [33] deployed transfer learning-based CNN to detect COVID-19 from X-Ray images.

Al-antari *et al.* [10] presented a simultaneous deep learning computer-aided diagnostic tool developed and based on the YOLO predictor for detecting and diagnosing COVID-19 lung disease from the entire chest X-Ray images. Their model was evaluated through five-fold tests for multi-class prediction problem by using two different chest X-Ray images. From the experimental results, the infected regions of COVID-19 from the whole X-Ray images were simultaneously detected and classified end-to-end through the CAD predictor, which achieved a good detection and classification accuracies greater than 90%. Moreover, the CAD deep learning approach showed greater reliability in assisting health care systems, patients, and physicians in delivering their practical validations. The CNN architecture proposed in the study was found to outperform 7 out of 12 established CNN architectures: AlexNet, GoogleNet, Vgg16, Vgg19, ResNet18, ResNet50, ResNet101, InceptionV3, InceptionResNetv2, SqueezeNet, Densenet201 and Xception. In another study, Majeed *et al.* [34] proposed an online attention module with 3D CNN to diagnose COVID-19 from CT scan images, and an accuracy of 87.5% was achieved.

The papers review indicated that CNN had attracted the research community’s attention in developing a diagnostic tool using CNN variants, most likely because of the images used to detect the COVID-19 from mostly X-Ray and CT scan images. Hybrid algorithms, transfer learning and automatic hyperparameter settings using optimization algorithms approaches are gaining momentum in detecting COVID-19. The review of the relevant studies clearly shows that room for improvement exists as 100% accuracy is yet to be achieved on the diagnosis of COVID-19 patients through CNN based on X-Ray and CT scan images. Therefore, further studies with improved performance are required to strengthen the diagnosis of COVID-19 patients.

## III. Covframenet: The Proposed Framework

In this section, an overview of the deep learning approach proposed in this study is presented. This overview is summarized using an architectural pipeline flow of the concepts and techniques applied. The datasets and the associated image pre-processing techniques adopted for this study are also detailed.

### A. Datasets

**FIGURE 1.**
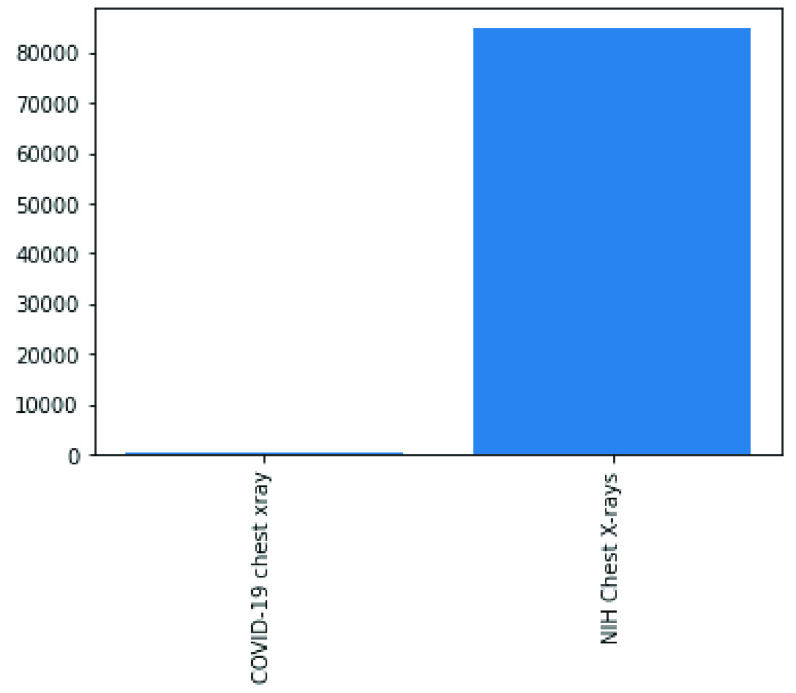
Comparison of the dataset sizes of the two major datasets (COVID-19 and NIH Chest X-Rays) used in this study.

The choice and the category of image samples applied to any CNN model are very important and require selecting an appropriate dataset. In this study, we decided to apply our CNN model to chest X-Rays or CT images which were outcomes of radiological imaging proven to have yielded a better diagnosis of COVID-19 [35]. Five (5) categories of datasets, publicly accessible, are employed to characterise the features and classification of the novel COVID-19 disease. These databases are the COVID-19 X-Ray images [36], the National Institutes of Health (NIH) Chest X-Ray Dataset [37], COVID-19 Radiography database [38], COVIDNet [39], Figure 1 COVID-19 Chest X-Ray [40], and Actualmed COVID-19 Chest X-Ray Dataset [41]. The most frequently accessed imaging is the chest X-Ray due to cost-effectiveness, although it presents a more challenging clinical diagnosis task than chest CT imaging. Hence, our combined approach of chest X-Rays or CT images and the use of publicly available datasets with large instances positioned our CNN model to achieve clinically relevant diagnoses.

The COVID-19 X-Ray dataset consists of COVID-19, MERS, SARS, and ARDS cases, represented as chest X-Ray or CT images samples. The database is accompanied by several attributes for each instance, which provides further details on the image sample. These fields include the number of days since the start of symptoms or hospitalization of patient (necessary for tracking multiple copies of the image taken per patient), sex, age, findings or outcome of the diagnoses, patient survival status, the view of the image presented (PA, AP, or L for X-Rays and Axial or Coronal for CT scans), modality (CT or X-Ray), clinical notes, and other important information. We obtained 363 instances of images and their accompanying metadata from the COVID-19 X-Ray database.

The second database is the National Institutes of Health (NIH) Chest X-Ray Dataset. This database is comprised of 112,120 X-Ray images which are of sizes 1024 x1024 with disease labels from 30,805 unique patients. The database provides samples of images and their diagnosed diseases and the disease region bounding boxes. Similar to the COVID-19 X-Ray dataset, this database also provides the following metadata about each instance: findings/diagnosis, type of disease diagnosed, age and gender of the patient, the view of the image and other details.

**FIGURE 2.**
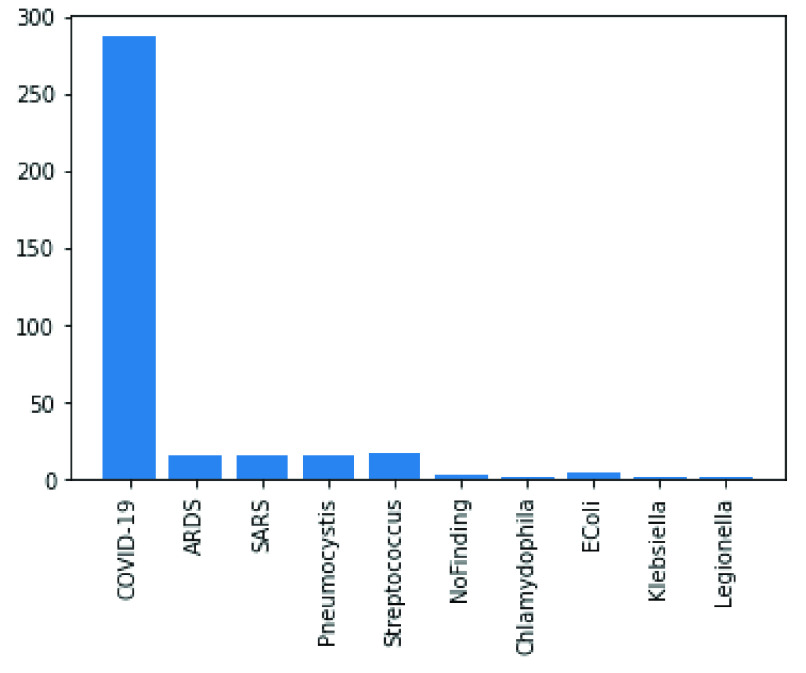
Classes of images available in the COVID-19 Chest X-Ray dataset.

**FIGURE 3.**
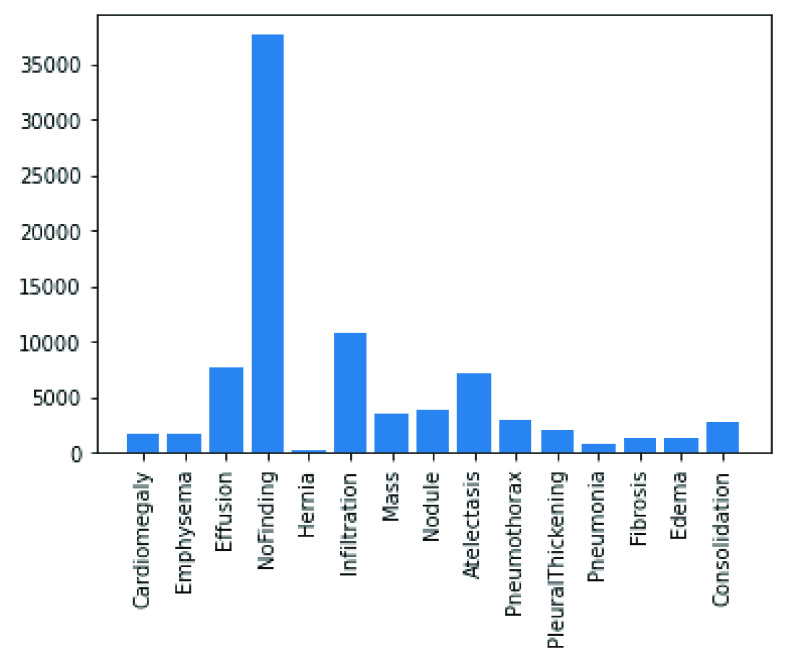
Classes of images available in the NIH Chest X-Ray dataset.

In the following figures, we have summarized the databases’ class distributions and sizes and present a combined chart of the two databases. Figure 1 shows the number of images in the COVID-19 chest X-Ray and NIH Chest X-Rays databases, which are 363 and 84823, respectively. Figure 2 reveals that the COVID-19 Chest X-Ray consists of ten (10) classes of images which include: COVID-19, 287 samples; Streptococcus, 17 samples; ARDS, 16 samples; SARS, 16 samples; Pneumocystis, 15 samples; E.coli, 4 samples; No findings or disease-free images, 3 samples; Chlamydophila, 2 samples; Legionella, 2 samples; and lastly Klebsiella, 1 sample. Similarly, there are 15 classes of images in the NIH Chest X-Rays databases (including the ‘No findings or disease-free label), which consist of Atelectasis, Consolidation, Infiltration, Pneumothorax, Edema, Emphysema, Fibrosis, Effusion, Pneumonia, Pleural thickening, Cardiomegaly, Nodule Mass and Hernia. The distribution of several instances across these classes of disease is as follows: No-Finding, 37645 samples; Infiltration, 10814 samples; Effusion, 7567 samples; Atelectasis, 7074 samples; Nodule, 3879 samples; Mass, 3415 samples; Pneumothorax, 2852 samples; Consolidation, 2766 samples; Pleural Thickening, 1984 samples; Cardiomegaly, 1715 samples; Emphysema, 1557 samples; Edema, 1352 samples; Fibrosis, 1219 samples; Pneumonia, 822 samples; and Hernia, 162 samples. These figures are charted in Figure 3.

**FIGURE 4.**
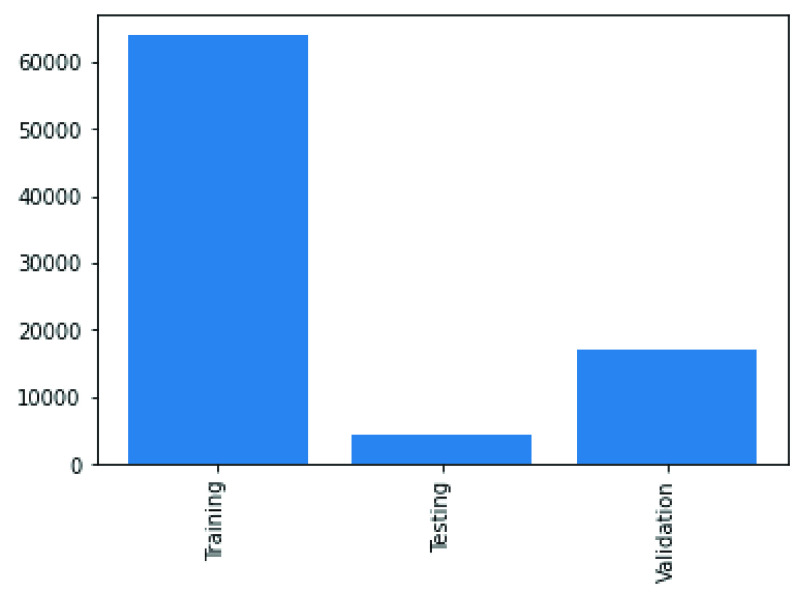
A combined graphing of distribution of images used for training, testing and validation as drawn from the COVID-19 and NIH Chest X-Ray datasets.

**FIGURE 5.**
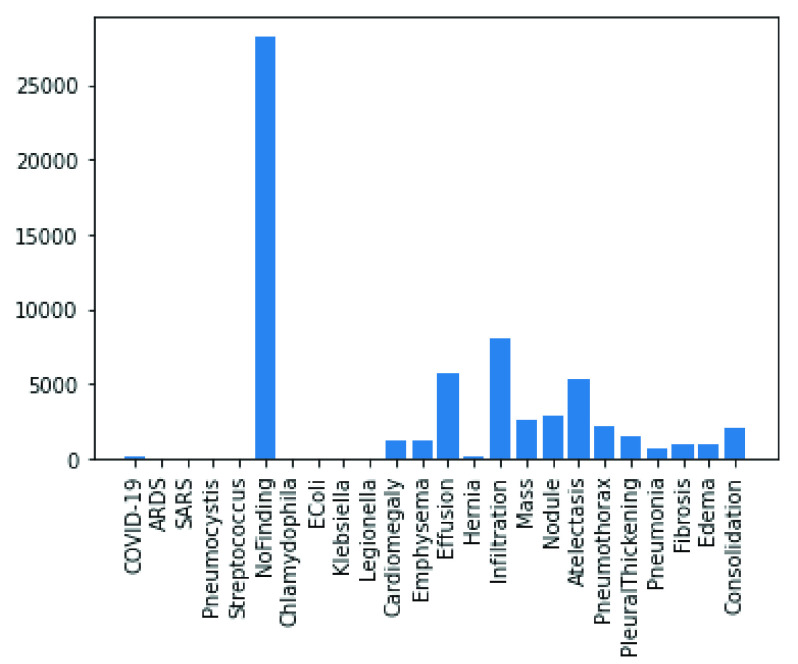
Distribution of training samples among classes of disease as drawn from the COVID-19 Chest X-Ray and NIH Chest X-Ray datasets.

**FIGURE 6.**
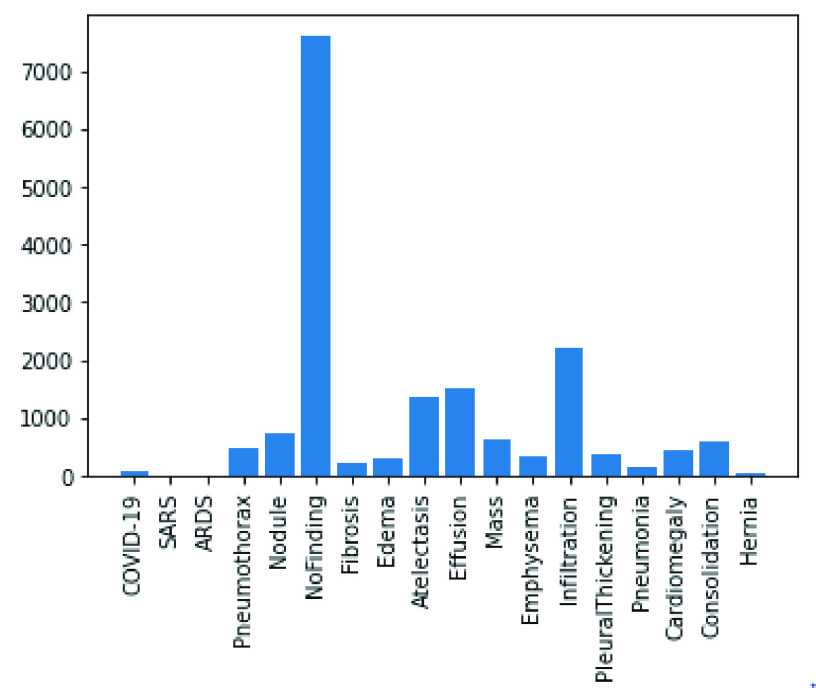
Distribution of validation samples among classes of disease as drawn from the COVID-19 Chest X-Ray and NIH Chest X-Ray datasets.

**FIGURE 7.**
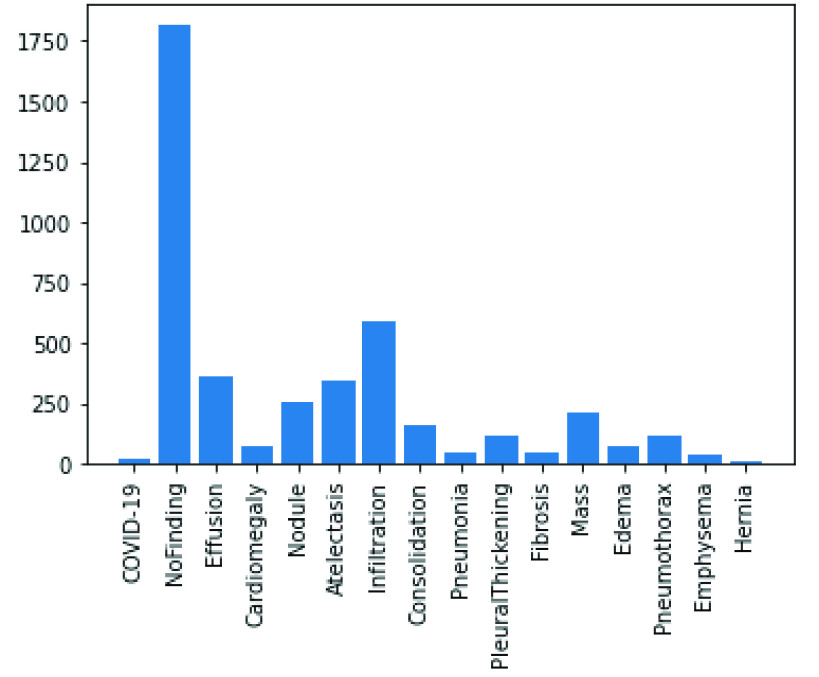
Distribution of testing samples among classes of disease as drawn from the COVID-19 Chest X-Ray and NIH Chest X-Ray datasets.

In the experimentation phase, the combined representation of the images from the two datasets were split into training, evaluation and testing categories and yielded 63887 samples for training, 17034 samples for validation and 4265 samples for testing. This is illustrated in Figure 4. A joint representation of the class distribution of images/samples across the two databases for training is shown in Figure 5. The combination yielded twenty-four (24) classes with the following number of samples in each class: No-finding or disease-free samples had 28222 images, Infiltration had 8017 images, Effusion had 5701 images, Atelectasis had 5373 images, Nodule had 2887 images, Mass had 2558 images, Pneumothorax had 2255 images, Consolidation had 2012 images, Pleural Thickening had 1511 images, Cardiomegaly had 1187 images, Emphysema had 1171 images, Edema had 1003 images, Fibrosis had 968 images, Pneumonia had 636 images, COVID-19 had 203 images, Hernia had 118 images, Streptococcus had 17 images, ARDS had 15 images, Pneumocystis had 15 images, SARS had 11 images, E.coli had 4 images, Chlamydophila had 2 images, Legionella had 2 images, and Klebsiella had 1 image. Meanwhile, a presentation of the splitting of the datasets into the evaluation and testing sets is captured in Figures 6 and 7.

We sourced for more COVID-19 samples from three (3) chest X-Ray databases namely: COVID-19 Radiography database [38], COVIDNet [39], Figure 1 COVID-19 Chest X-Ray [40], and Actualmed COVID-19 Chest X-Ray Dataset [41]. After combining these supporting datasets, we obtained 69918 image samples for training, 17319 samples for validation, and 4587 samples for testing. Considering the level of noise, distortion, or anomalies associated with some of the images accessed from the publicly available databases, this study pre-processed all the samples. This was achieved by applying some standard image pre-processing techniques to our images. The next section details this approach.

### B. Image Pre-Processing

This study adopted some image pre-processing techniques to enhance the performance of the proposed deep learning model. This approach is also seen in related studies [42], [43], [44], [45], which have encouraged the application of inputs/samples to appropriate pre-processing techniques/algorithms. Image processing, which uses algorithms to perform image processing on digital images, is categorized into analogue image processing and digital image processing. The pre-processing techniques aim to improve the features in the image through image enhancement and the suppression of unwanted distortions, thereby yielding an improved image/input for the deep learning model. In this study, we applied our samples to the following pre-processing techniques after reading or loading images into the buffer:

• Image resizing: Due to the heterogeneity of the databases and variations in the sizes of images, we resized the images into $220\times 220$ sizes. Such resizing operation allowed for decreasing the total number of pixels from $888\times 882$ and $1024\times 1024$ for COVID-19 X-Ray and NIH Chest X-Ray datasets $220\times 220$ for both.
• Removal of noise (denoise): Image denoising can present a challenging procedure arising from the operation of estimation of the original image with the hope of eliminating noise. For instance, one might be interested in removing any of the following noises from an image: Poisson noise, salt and pepper noise, Gaussian noise, and speckle noise. In this study, we attempted to eliminate/remove noise from our image samples using the Gaussian Blur technique since study [46] showed that the technique is relevant in images with high noise. We used a Gaussian filter by applying our images to the function cv2.GaussianBlur using kernel size of $5\times 5$ and zero (0) for both the standard deviation for both the $x$ and $y$ directions.
• Morphology (smoothing edges): As a pre-processing operation, we applied the morphology operation to our samples before applying segmentation to our images. This enabled us to extract image components that were useful in the representation and description of region shape. This operation (morphological smoothing) aimed to remove bright and dark artefacts of noise through an opening and closing operation. The output of this phase yielded images whose edges were smoothened for easy detection.
• Segmentation: It is well-known that image segmentation allows for the partitioning of an image into multiple image objects or segments appearing as different categories of pixels such that a similar category constitutes a segment. This study applied this technique to enhance detecting image objects that support feature extraction, thereby obtaining meaningful results. We achieved this by using thresholding methods, leaving out other methods such as edge detection-based techniques, region-based techniques, clustering-based techniques, watershed-based techniques, partial differential equation-based and artificial neural network-based techniques. Using the thresholding method, we used the THRESH_BINARY_INV thresholding style of OpenCV and a maxVal of 255, representing the value to be given if the pixel value is more than the threshold value. The computation of THRESH_BINARY_INV is as shown in (1).
\begin{align*} \mathrm {dst}\left ({x,y }\right)=\begin{cases} \displaystyle 0,& \mathrm {src}\left ({x,y }\right)>\mathrm {thresh} \\ \displaystyle \mathrm {maxVal},& \mathrm {otherwise} \\ \displaystyle \end{cases}\tag{1}\end{align*}

The second parameter to the maxVal is the retVal as used in our thresholding technique. Otsu’s method is widely reported to yield interesting results and is also suitable for distinguishable foreground and background [47]. The use of this method was inferred from the value we set for the retVal, which is the THRESH_OTSU. This allowed for automating the process of calculating the threshold value from the image histogram. Thus far, we have filtered our image samples with a $5\times 5$ Gaussian kernel to remove the noise and then applied Otsu thresholding.

Furthermore, we applied the dilate operation on the image to enlarge the foreground and find the sure background area. Also, to find the sure background area in the image, we applied the distance transform operation to represent a binary image so that the value of each pixel was replaced by its distance to the nearest background pixel. Hence the threshold segmentation was applied to divide the image into regions of object and background. Our thresholding segmentation was completed by applying global thresholding, which uses any appropriate threshold value of $T=$ kept constant for the whole image so that the output image is obtained from the original image as seen in (2) [48].

\begin{align*} q\left ({x,y }\right)=\begin{cases} \displaystyle 1,& \text {if}~p\left ({x,y }\right)>T \\ \displaystyle 0,& \mathrm {if}~p\left ({x,y }\right)\le T \\ \displaystyle \end{cases}\tag{2}\end{align*}

The resulting images from all the pre-processing techniques above were then passed as input into the CNN model described in the following subsection.

### C. The CNN Architecture

**FIGURE 8.**
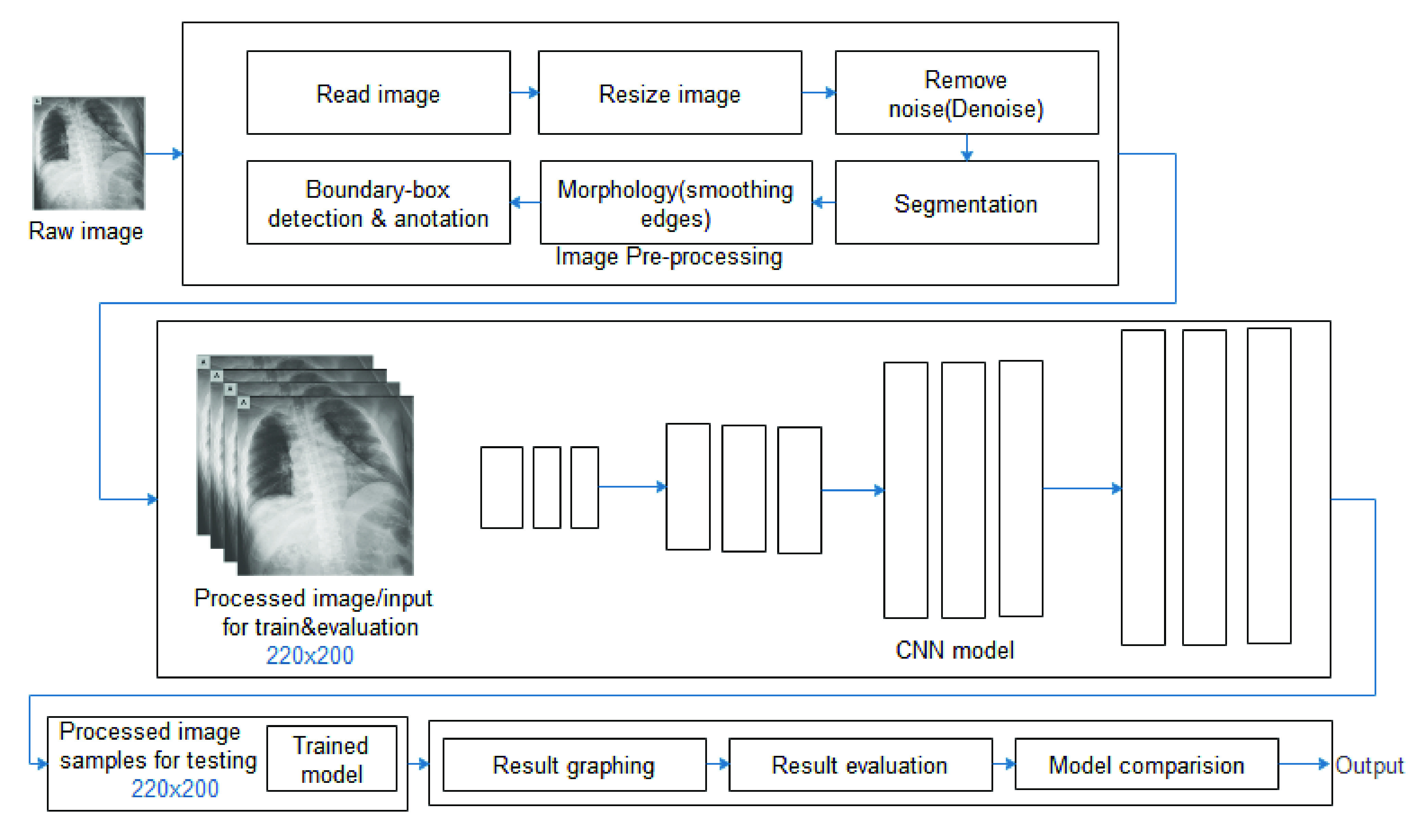
The CovFrameNet framework showing image/input pre-processing, feature detection, sample classification and post-classification processing.

The proposed CNN model is a component of a complete framework in Figure 8, representing the pipeline flow of techniques used in this study. The architectural pipeline shown in the figure reads in the sample images from the databases, and then pre-processing techniques described in the previous subsection were applied sequentially. Furthermore, the resized and improved image samples were split into training and validation sets based on the illustration shown in the previous subsection. After that, the CNN model was applied to the input samples for training and validation. The trained model was then applied to the test set of images for prediction, and then the result of the classification was output for performance evaluation.

**FIGURE 9.**
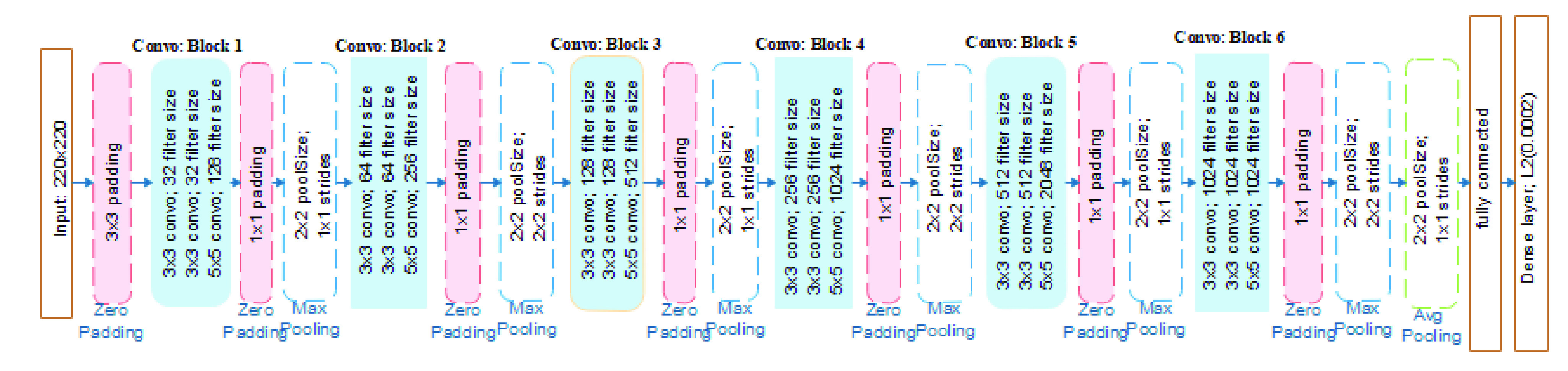
The architecture of the proposed convolutional neural network (CNN) for feature detection and classification COVID-19 disease from chest images.

CNN is the most widely used deep learning model for image recognition. Medical image recognition tasks have largely benefited from the field of computing. CNN consists of a convolution layer that extracts the features of the image and a fully connected layer that determines which class the input image belongs to – the classification of the input image. In Figure 9, we present the architecture of the proposed CNN model designed and applied to our datasets in this study. The architecture of the model follows the form of Conv-Conv-Conv-Pool-Drop-Conv-Conv-Conv-BatchNorm-Pool-Drop-Dense(relu)–BatchNorm-Drop, with many filters modeled as 32(3, relu)–32(3, relu)-128(5, relu)–2(2)-64(3, relu)–64(3, relu)-256(5, relu) and so on. For the classification purpose, we applied the SoftMax function to the model’s feature detection phase. This allowed for a multiclass classification as against the binary classification in our case.

The proposed CNN model benefits from some deep learning regularization techniques, demonstrating the capacity to combat the overfitting issue. Overfitting is the situation when a model learns the training data excellently but falls short of generalizing well when some other data is exposed to it. Regularization techniques such as L2 and L1, dropout, data augmentation, and early stopping have been widely reported to enhance the performance of deep learning models [49], [50]. Therefore, this study experimented with some techniques to ensure optimal performance of the proposed deep learning (CNN) model. Hence, we did not just hope to improve performance but also to enable our model to generalize well. A model failing to generalize well will show validation error increasing while the training error steadily decreases. In this study, we applied our work to the most common regularization technique L2, which is also referred to as “weight decay”, to eliminate overfitting. L2 values range between 0 and 0.1 with examples as 0.1, 0.001, 0.0001, and are in logarithmic scale. We, therefore, hope to reduce our model’s training error [51], [52] by applying this technique. For instance, the Inception V3 model experimented with a value of 0.00004 [53]. We discovered that it was suboptimal and instead experimented with 0.00005. In addition to the use of L2, we also demonstrated early stopping to stop our model from continuing training when it had attained its optimal performance. This regularization concept is another widely used technique in deep learning to stop training when generalization error increases. The proposed CNN model was also experimented with to use a dropout layer at the rate of 0.5.

## IV. Experimentation

In this section, the COVID-19 chest X-Ray and NIH chest X-Ray datasets described in the previous section are applied to the CNN model for training. Furthermore, the performances of the CNN model on multiclass classification are evaluated. The environment for the experimentation and the outcome of the pre-processing techniques are also described.

### A. Configuration Environment

All our experiments were carried out on Google’s Colab environment with the following configurations: 2-core Intel(R) Xeon(R) CPU @ 2.30GHz, 13GB memory and 33GB hard drive; and GPU Tesla P100-PCIE-16GB.

### B. Application of Image Pre-Processing

**FIGURE 10.**
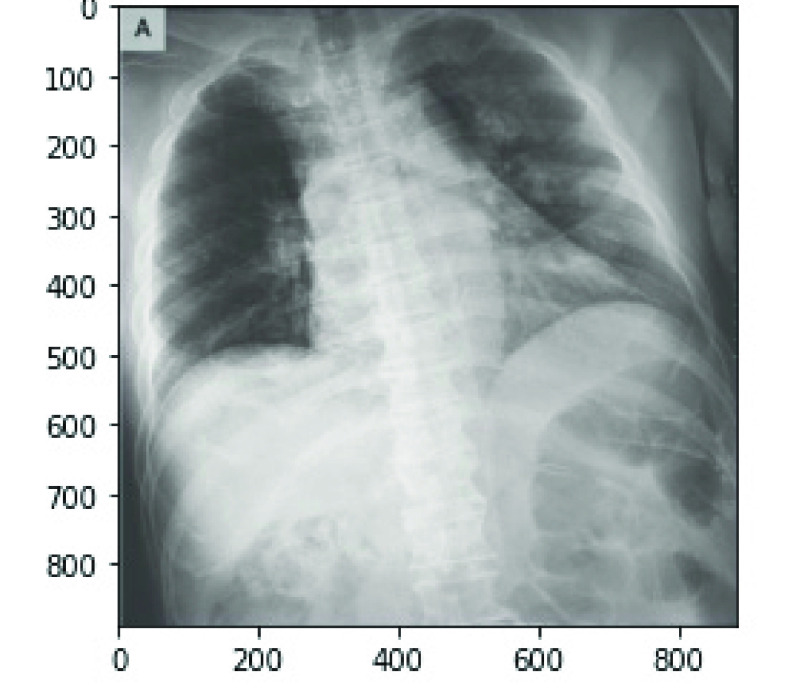
A sample of raw image of size $888\times 882$ from the COVID-19 Chest X-Ray dataset.

**FIGURE 11.**
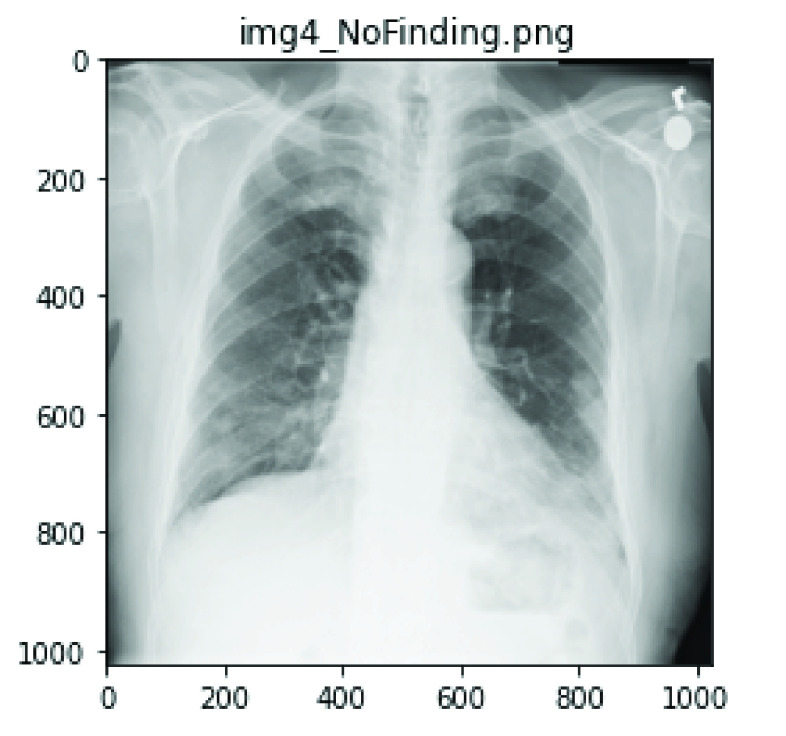
A sample of the raw image labeled ‘No Finding’ of size $1024\times 1024$ from the NIH Chest X-Ray dataset.

**FIGURE 12.**
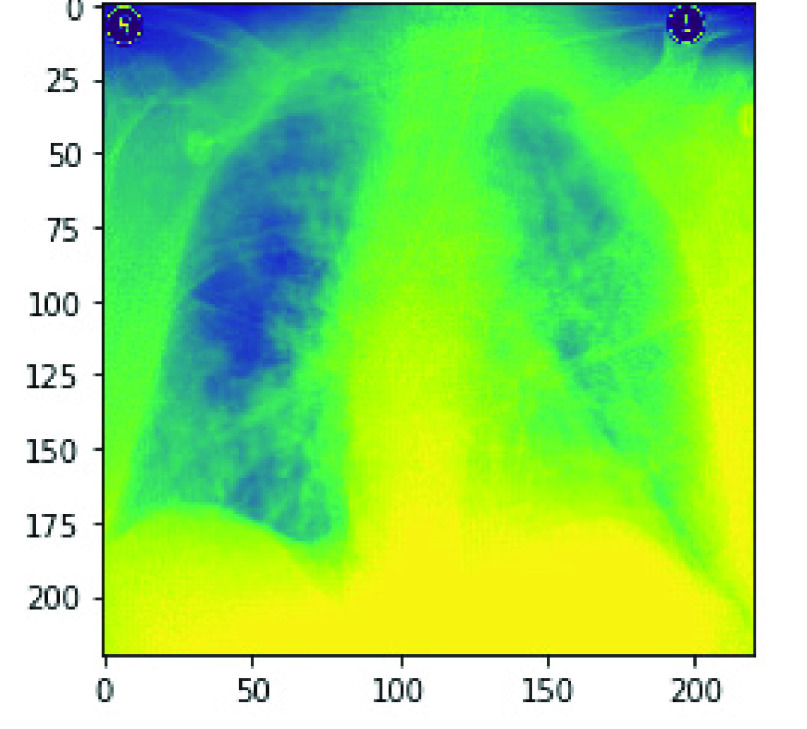
A resized sample from $888\times 882$ to $220\times 200$ in the COVID-19 Chest X-Ray dataset.

The pre-processing techniques applied to our input images/samples were extensively discussed in previous section. Therefore we aim to present the outcome of the application of those techniques on our datasets. The first operation applied was the resizing of images from the high resolution of $888\times 882$ and $1024\times 1024$ to a collective size of $220\times 220$. This was necessary to allow the datasets sourced from different platforms to feed into our model effectively as a fixed size. Figures 10 and 11 show the original image samples from COVID-19 and the NIH chest X-Ray datasets, respectively, and the outcome of the resizing of the operation shown in Figure 12.

**FIGURE 13.**
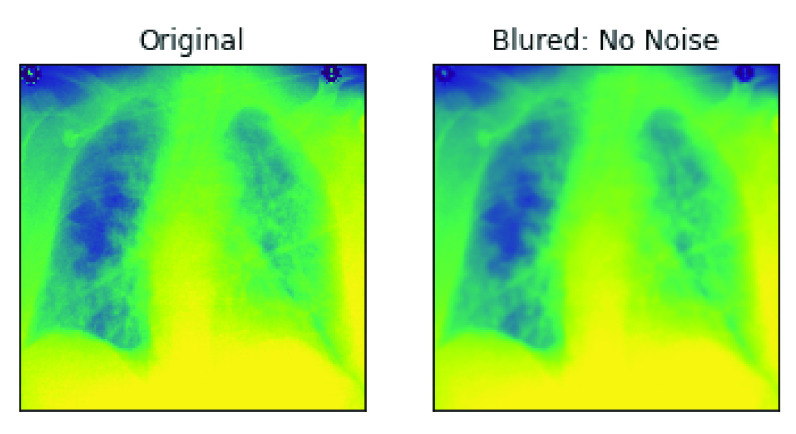
An illustration of image pre-processing task carried out on samples through the denoising and blurring effects.

**FIGURE 14.**
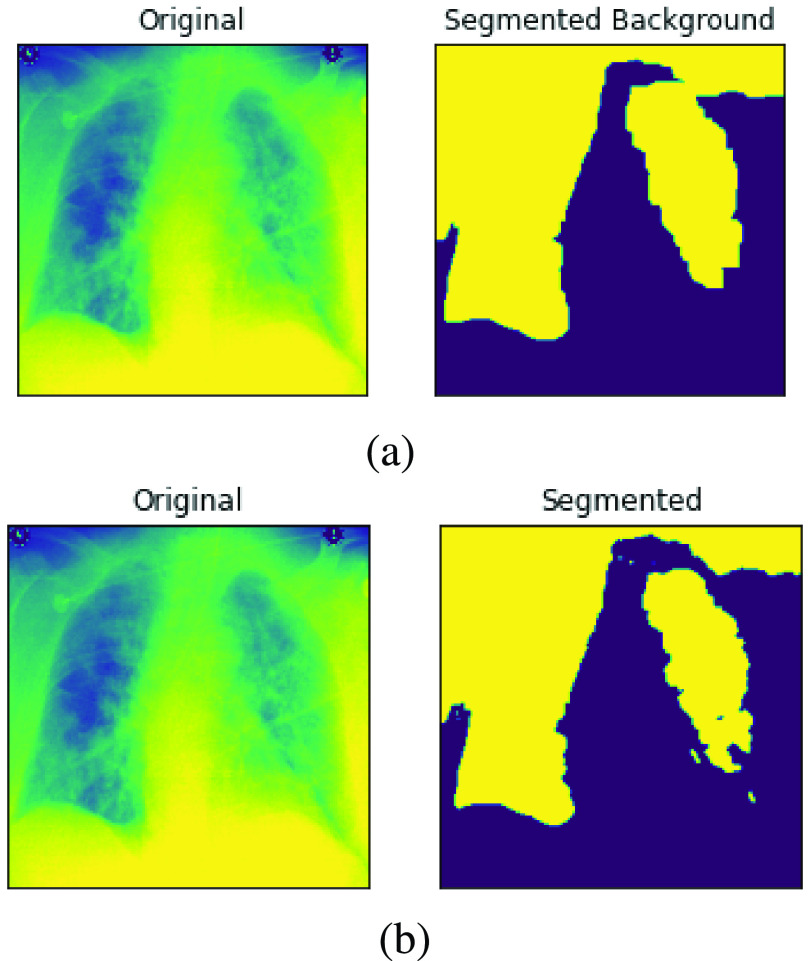
a. An illustration of image pre-processing task aimed at segmenting samples b. An illustration of image pre-processing task aimed at extracting segmented background from samples.

One major pre-processing operation carried out on our input sets was removing noise as described in the previous section. The approach taken in this study was to blur the image samples as a measure to clean and denoise them. Hence, in Figure 13, a pair of samples resulting from the un-denoised and denoised image is captured and shown. Furthermore, to demonstrate the segmentation operation carried out on the image samples by this study, we have also presented output from such operation. In Figures 14 (a-b), a pair of samples of images with segments and background extracted are presented. The pair of images in Figure 14a shows the original image and the outcome of the segmented images, while that of Figure 14b shows the original image and its extracted background. These operations allow for an easier understanding of what is in the image and enable an easier analysis of each part. In addition, the segmentation operation on our medical X-Ray images were segmented within the image typically for further investigation.

Bounding boxes is one of the most interesting operations supporting image annotation in deep learning models. This proves useful in object classification in images and even for further localization tasks. Whereas image is aimed at assigning a class label to an image, object localization allows for creating bounding boxes around recognizable objects in the image. The model’s target to classify and obtain positions of objects in the image is referred to as object detection or object recognition. Drawing bounding boxes can be achieved using deep learning models or other algorithms. For instance, to describe the location of some targeted diseases in our input images, we drew a bounding box as a rectangular box that can be determined by the $x$ and $y$ axis coordinates in the upper-left corner and the $x$ and $y$ axis coordinates in the lower-right corner of the rectangle. This operation allows for easily annotating our samples for convenient recognition by CNN model.

**FIGURE 15.**
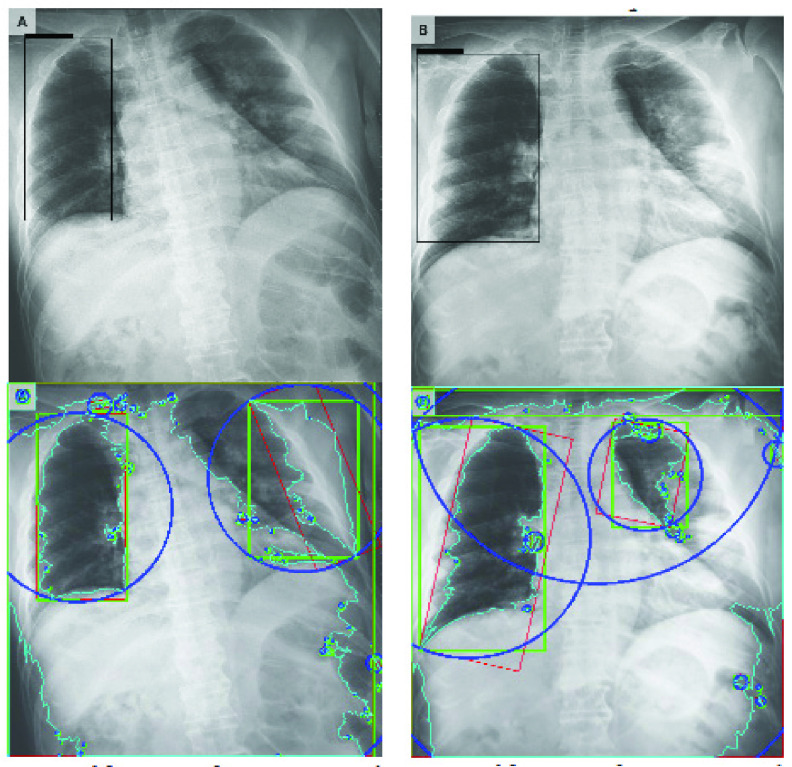
Samples of images and their respective findings with expert annotation as extracted from the NIH Chest X-Ray dataset. The bounding boxes show the suspected regions of COVID-19 in PA view. Below is a capture of the contours in the image samples.

**FIGURE 16.**
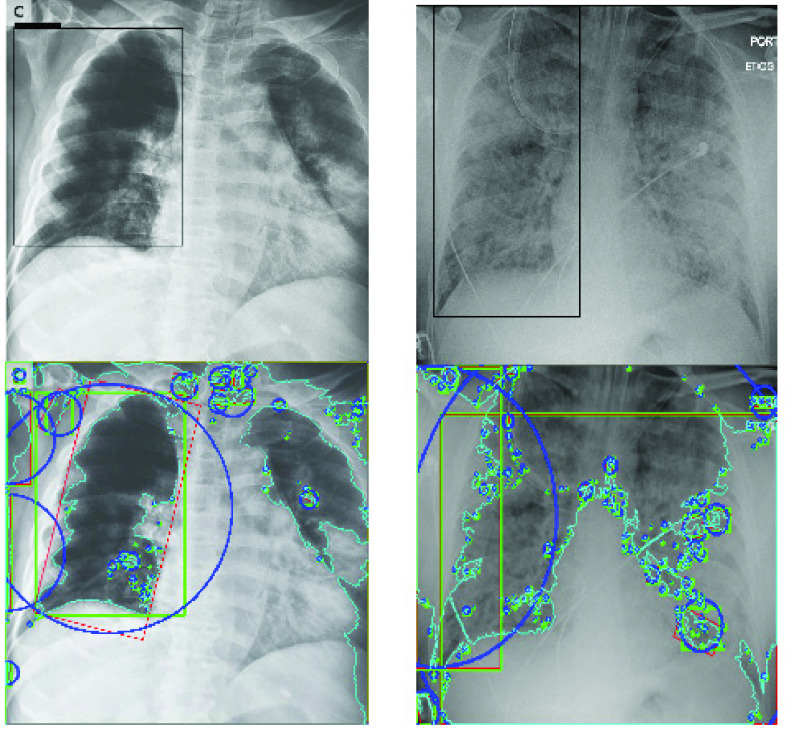
Samples of images and their respective findings with expert annotation as extracted from the NIH Chest X-Ray dataset. The bounding boxes show the suspected regions of COVID-19 and ARDS in PA views. Below is a capture of the contours in the image samples.

Figures 15 (a-b) and 16 (a-b) show the bounding boxes (using black colour) locating the position of the labelled disease on the image and their corresponding contours. In addition to drawing bounding boxes around input, we detected contours both in the bounding box and those outside it. In each case of images in Figures 15 and 16, the upper image represents the bounding box localizing the presence of the disease while the lower figure represents the contours in each image.

Contours allow for identifying the shapes of objects within an image and are recognized through lines of curves joining all the continuous points with similar colour or intensity. This technique provides support for object detection and recognition. In this study, to extract the contours as shown in the images below those with the bounding boxes, we carried out the following: first thresholded each image and then found all the contours in each image; with each contour, we drew a bounding rectangle in green colour; then got a minimum rectangle area and converted all coordinates floating-point values to integer, and drew a red ‘nghien’ rectangle; furthermore, we got the minimum enclosing circle and converted all values to an integer to draw the circle in blue; then finally drew all contours on each image.

The proposed CNN model receives grayscale images as its input, and experiments were performed with multiclass classifications. Table 1 shows the detection classes for each classification and their distribution in both datasets. Meanwhile, for each experiment carried out, we trained the model for 50 epochs and 1310 steps.

**TABLE 1:** Classes for Multiclass Classifications for the COVID-19 and National Institutes of Health (NIH) Chest X-Ray Datasets

No.	COVID-19 Multiclass	NIH Multiclass
1	COVID-19	COVID-19
2	MERS	Atelectasis
3	SARS	Hernia
4	ARDS	Mass
5	No-Finding	No-Finding
6	Streptococcus	Consolidation
7	E.coli	Infiltration
8	Pneumocystis	Pneumothorax
9	Chlamydophila	Edema
10	Legionella	Emphysema
11	Klebsiella	Fibrosis
12		Effusion
13		Pneumonia
14		Pleural thickening
15		Cardiomegaly
16		Nodule

### C. Evaluation Metrics

To evaluate the performance of the proposed model, we computed accuracy, sensitivity, specificity, precision, recall, F1-score, Cohen’s Kappa, ROC AUC, and confusion matrix. The following paragraphs briefly outline the metrics and their relevance to our classification of novel COVID-19 disease. The metric precision checks what proportion or quantity of positive identifications achieved by a model was correct and given by (3).

\begin{equation*} \mathrm {Precision}=\frac {\mathrm {TP}}{\left ({\mathrm {TP}+\mathrm {FP} }\right)}\tag{3}\end{equation*}

On the other hand, recall checks the number of actual positive cases in our datasets which the proposed CNN model was able to identify correctly. This is given by (4).

\begin{equation*} \mathrm {Recall}=\frac {\mathrm {TP}}{(\mathrm {TP}+\mathrm {FN})}\tag{4}\end{equation*}

Evaluating the effectiveness of our CNN model requires that we examine its performance in terms of precision and recall, hence the need to compute these metrics. Furthermore, we examined another metric known as the F1 Score. This metric expressed the balance between the precision and the recall described above and helped us decide whether the performance of our model was based on precision and recall. We give the equation for the F1 score in (5).

\begin{equation*} \mathrm {F1-Measure}=\frac {\left ({\mathrm {2\ast Precision\ast Recall} }\right)}{\left ({\mathrm {Precision+Recall} }\right)}\tag{5}\end{equation*}

In this study, we chose an under-utilized, though effective, multiclass classification metric known as Cohen’s Kappa. This metric is robust in handling imbalanced class problems, as may be seen in our datasets. In a multiclass classification problem, this metric provides a broader view of the performance of a classification model compared to accuracy in (6) or precision/recall. The metric is represented in (7).

\begin{align*} \mathrm {Accuracy}=&\frac {\mathrm {TP+TN}}{\mathrm {(TP+TN+FP+FN)}} \tag{6}\\ \mathrm {K }\equiv&\frac {\mathrm {p}_{\mathrm {o}}-\mathrm {p}_{\mathrm {e}}}{1-\mathrm {p}_{\mathrm {e}}}=1- \frac {1-\mathrm {p}_{\mathrm {o}}}{1-\mathrm {p}_{\mathrm {e}}}\tag{7}\end{align*}

The receiver operating characteristic (ROC) curve expresses the performance of the classification model using a graphical approach and does these at all classification thresholds. It can achieve this by graphing the True Positive Rate (TPR) and False Positive Rate (FPR). The metric gives a summary of the performance of a classifier over all possible thresholds. Similar to the ROC is the area under the ROC curve (AUC), which examines the entire two-dimensional area underneath the entire ROC curve that covers (0,0) to (1,1). This metric is effective at checking the proper/wellness and quality of our model’s prediction performance. Finally, we have the confusion matrix. Whereas the accuracy of a model may seem appealing in some sense, it is limited by its inability to give details of the performance of the classification model. On the other hand, the confusion matrix presents this detail by unambiguously presenting the prediction result.

## V. Results and Discussion

This section presents the performance of the CNN architecture and a comparative analysis of the model with similar studies. We experimented with the proposed CNN model on the datasets using some variation of hyperparameters. For instance, we investigated the model’s performance when SGD and Adam optimizers are applied to the model and plotted the model’s output. Furthermore, we experimented on our proposed model to discover the effect of two different values for the L2 (weight decay) regularization technique.

**FIGURE 17.**
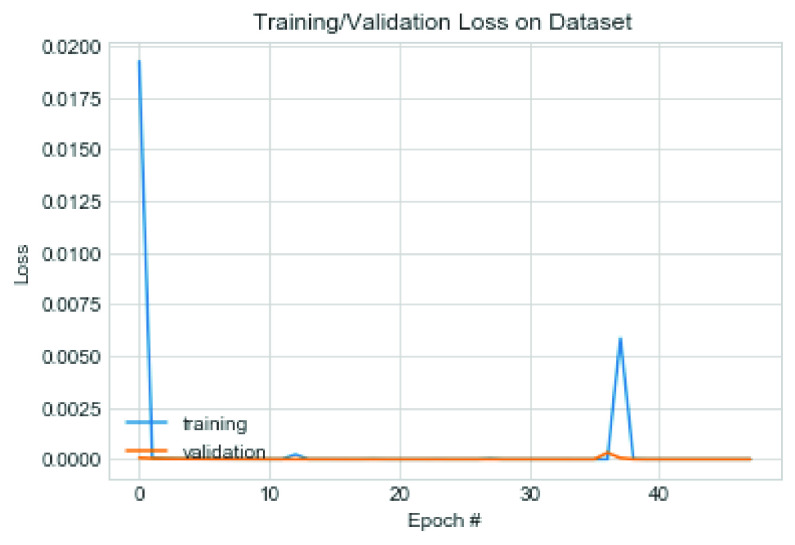
Pattern of change in loss function of training and validation on the combined dataset using Adam optimizer.

**FIGURE 18.**
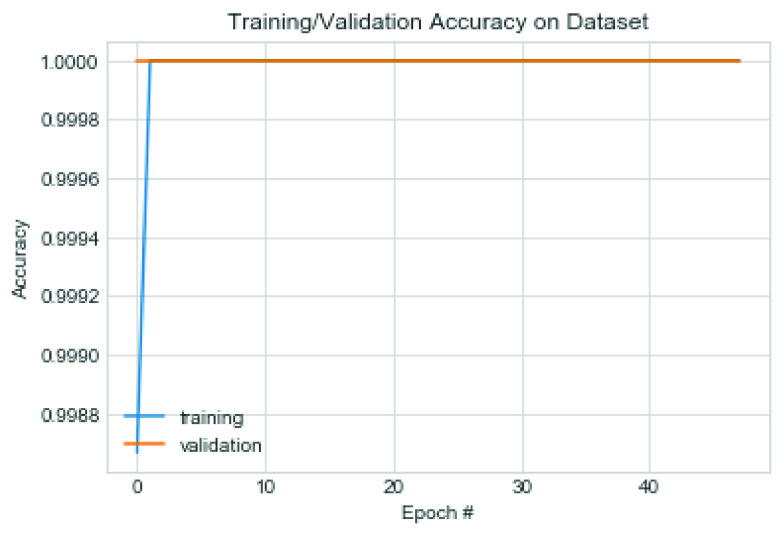
Pattern of change in accuracy of training and validation on the combined dataset using Adam optimizer.

**FIGURE 19.**
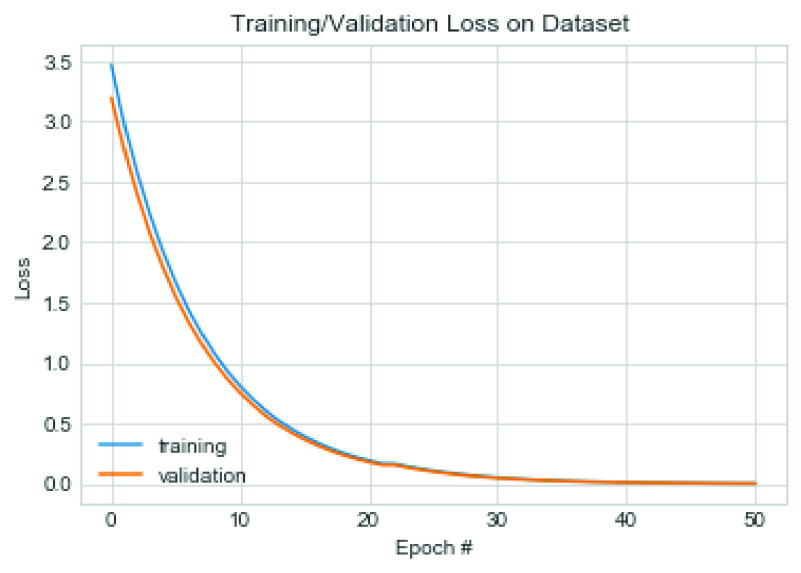
Pattern of change in loss function of training and validation on the combined dataset using SGD.

**FIGURE 20.**
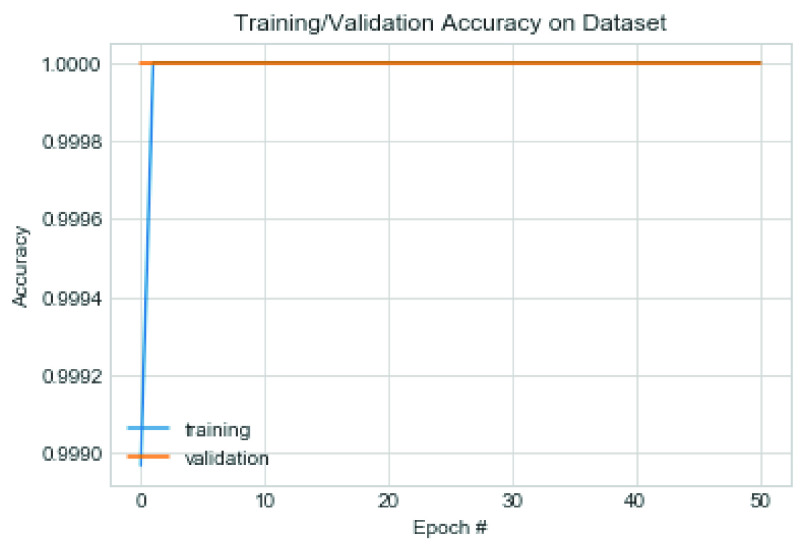
Pattern of change in accuracy of training and validation on the combined dataset using SGD optimizer.

The first set of experiments used the Adam optimization algorithm and weight decay (L2) value of 0.0002. Figure 17 captures the model’s performance in terms of the loss function, while Figure 18 shows the trajectory of the accuracy for both training and validation cases. Note that the configuration of the Adam optimizer is as follows: learning rate $=0.001$, beta $1=0.9$, beta $2=0.999$ and epsilon $=1\text{e}$-8. Similarly, in the second experiment, we experimented using the SGD optimizer with the following configuration: learning rate $=0.01$, decay $=1\text{e}$-6, momentum $=0.9$ and nesterov = True. The value of 0.0005 was used for the L2 regularization technique. The performance of the model was also examined, and we found that although the accuracy remained close to that of the Adam optimizer, there was, however, a difference in the loss values trajectory. Figures 19 and 20 capture the performance of the model on the training and validation for loss function and accuracy. We also exposed the trained model to the test dataset under the same configuration.

The result showed that the CNN model using SGD learnt the problem effectively as the loss values for training and validation progressively dropped. Although a slight dispersion appeared in their loss values between 0–30 epochs, the values significantly closed between epochs 30-50. The combined performance of the training and loss supports the argument that the proposed CNN model significantly learns and detects COVID-19 features. On the other hand, we observed that Adam’s loss values for training and validation showed some irregularity. This irregularity was observed in epochs 30–38 while it normalized around 38–50 epochs. These observations showed that the application of SGD outperformed Adam in the CNN architecture proposed in this study. The experimental results of the multiclass classification for two (2) experimental cases showed more than 99% accuracy.

In Table 2, we listed the performance of our model for the experiments carried out in comparison with similar models adapted to the purpose of classification COVID-19 disease.

**TABLE 2:** Summary of Result Obtained by the Proposed Model

Authors	Specificity	Sensitivity	Precision/Recall	F-score	AUC	Accuracy
Adam	1.00	–	0.85/0.85	0.90	0.50	1.00
SGD	1.00	–	0.85/0.85	0.90	0.50	1.00
[43]	0.80	0.90	–	0.90	0.85	0.85
[54]	0.10	0.99	–	–	–	0.99
[63]	1.0	0.99	1.0	0.99	–	0.99
[64]	1.0	0.99	1.0	0.99	–	0.99

The result obtained in Table 2 showed that our system achieved 1.00, 0.85, 0.85, 0.90, 0.50, and 1.00 for specificity, recall, precision, F-score, AUC, and accuracy for phase one of the experiment. On the one hand, in the second experiment carried out, our model yielded the following: 1.00, 0.85, 0.85, 0.90, 0.50, and 1.00 for specificity, recall, and precision, F-score, AUC and accuracy, respectively. The proposed model attained 85% precision and recall, making it useful for the proposed task, eliminating unnecessary false alarms. F1 measure is relevant if we are looking to select a model based on a balance between precision and recall, and is the harmonic mean of precision and recall and gives a better measure of the incorrectly classified cases than the accuracy. As a result, the value of 0.9 for our F1-score shows the performance of our model even when there are imbalanced classes, as is the case in most real-life classification problems.

**FIGURE 21.**
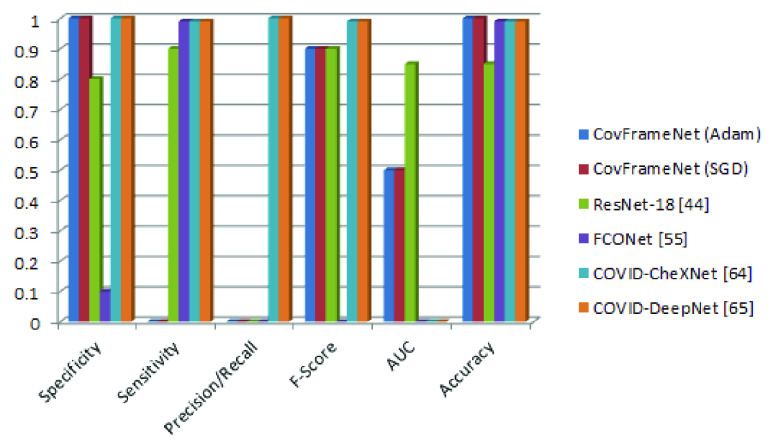
Comparative analysis of the performance of the proposed architecture with similar studies using Specificity, Sensitivity, Precision, Recall, F-score, and AUC.

Figure 21 illustrates the relevance of the outcome of this study when compared with related CNN architectures designed for the detection of COVID-19. The proposed CovFrameNet model is seen to strongly compete with state-of-the-art models using specificity, precision, F-score, AUC, and accuracy as metrics for the comparative analysis. We argue that the accuracy of CovFrameNet outperforms that of its similar structure – ResNet. Moreover, we see CovFrameNet competing with ResNet, FCONet, COVID-CheXNet and COVID-DeepNet using F1-score. Although the proposed CovFrameNet falls short in AUC, precision, and recall compared with ResNet, it demonstrates a competitive result considering specificity.

In Table 3, we list the performance of our model compared to other similar models used for the detection of COVID-19, with emphasis on the accuracy of the concerned models.

**TABLE 3:** Comparing the Contributions and Performances of the Proposed Study With Similar Approaches

Authors	Contribution/Approach	Dataset	Accuracy
Proposed approach	Deep learning architecture for effective classification of COVID-19	COVID-19 and NIH Chest X-Ray datasets	100.00%
[16]	VGG19, Mobile Net, Inception, Xception, and InceptionResNet v2		93.48%
[14]	DarkCovidNet	Raw chest X-Ray images	87.02%,
[17]	CoroNet		89.60%
[38]	COVID-Net		92.40 %
[43]	COVID-Net and ResNet-18	COVID-ChestXRay dataset	85.00% and 100.00% respectively
[55]	Deep learning-based Decision-tree classifier	COVID-ChestXRay dataset	95%
[54]	Fast-track COVID-19 classification network (FCONet)	3993 Chest CT images privately collected	99.87%
[56]	nCOVnet	Chest X-Ray Images	97.00%
[63]	COVID-CheXNet	–	99.9%
[64]	COVID-DeepNet	–	99.9%
[65]	SVM+IOT		95.0%

Considering the performance of our model from Table 3 compared with other similar studies, we conclude that this study outperforms state-of-the-art deep learning models aimed at detecting and classifying the novel Coronavirus (COVID-19). This is clear from the performances of similar studies when compared with this study. We note that only Ko *et al.* [55] and Panwar *et al.* [57], who used the fast-track COVID-19 classification network (FCONet) and nCOVnet, respectively, have their models’ performances competing with our model. Therefore, this study has successfully advanced research in the areas of detection and classification of COVID-19 using deep learning models.

**FIGURE 22.**
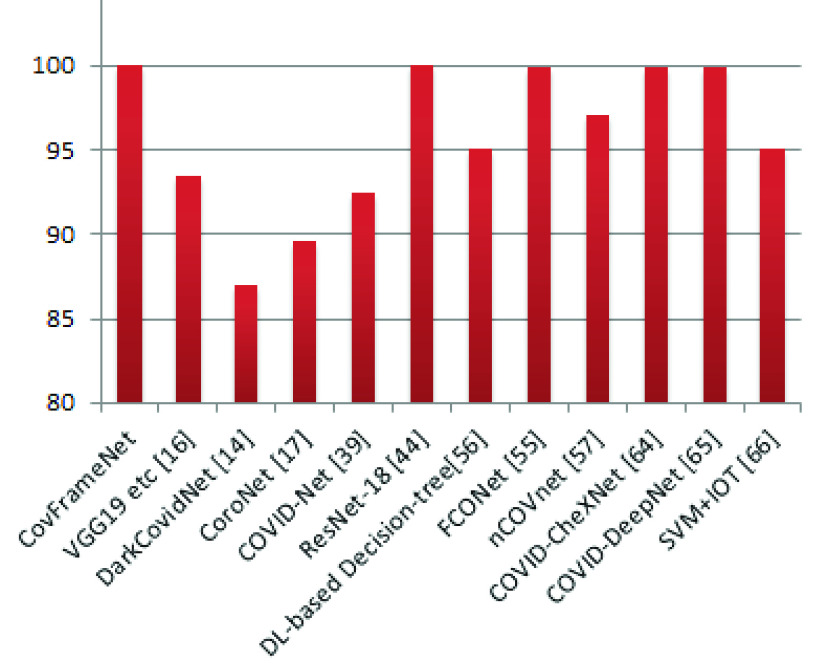
Comparative analysis of the performance of the proposed architecture with similar studies based on the accuracy.

As earlier noted, the proposed CNN model is patterned after ResNet architecture, and the result obtained, as shown in Figure 22, confirms that the former outperformed the latter. Similarly, we note that popular networks such as VGG19 and other related networks designed to detect COVID-19 are seen to lag behind the proposed CovFrameNet. This further demonstrates that CovFrameNet network architecture is well suited for extraction COVID-19 features and further classification purposes.

In summary, the findings in this study show that deep learning models can sufficiently detect the presence of COVID-19 from digital chest X-Rays. To minimize the high risk of bias in the study, we ensured that considerably large samples of COVID-19 cases were applied. This was also complemented by using publicly available datasets in combination with additional new data to overcome overfitting. Also, the data were widely pre-processed using combinatorial methods to ensure inputs to the CNN model were acceptable. To promote reproducibility of our approach, our algorithm and implementation are publicly available at https://github.com/NathanielOy/covid19cnn

## VI. Limitations of the Study

The CNN architecture proposed in this study was designed based on the authors’ expertise in neural network architectures. Although the number of parameters was memory-demanding, this could be further scaled down by optimising hyperparameters, which will eliminate operations that do not significantly contribute to the algorithm.

## VII. Conclusion

In this paper, a deep learning model based on CNN was designed and implemented to detect and classify the presence of COVID-19 in chest X-Rays and CT images. The study’s main contribution involves applying selected image pre-processing techniques and the design of CNN architecture both encapsulated in a deep learning-based framework. The proposed framework pipelined the entire procedure in a manner to enhance the performance of the classification. Furthermore, we investigated the performance of the proposed model by juxtaposing the use of optimizer between the popular Adam and SGD. The result revealed that our model achieved 100% accuracy in classifying the novel coronavirus (COVID-19) using SGD. The outcome of this study showed that a CNN-based solution might be adopted in pre-screening suspected cases and confirmation of RT-PCR-based detected cases of COVID-19. The training of the CNN model was partly impaired by the availability and access to COVID-19 images. This study’s future research direction is recommended to explore the high volume of chest X-Ray images emerging from new cases for fine-tuning the CNN architecture. Furthermore, it will be interesting to see the deployment of our trained CNN model to both web and mobile applications for clinical utilization.

## Conflict of Interests

The authors declare that they have no known competing financial interests or personal relationships that could have appeared to influence the work reported in this article.
